# Precopulatory oral sex contact plays an important role in copulatory success in a cryptic desert beetle

**DOI:** 10.1002/ece3.6595

**Published:** 2021-08-25

**Authors:** Xinghu Qin, Jinshu Yang, Jingchuan Ma, Thomas Ryan Lock, Guangjun Wang, Zehua Zhang

**Affiliations:** ^1^ Institute of Plant Protection Chinese Academy of Agricultural Sciences Beijing China; ^2^ School of Biology University of St Andrews St Andrews UK; ^3^ Scientific Observation and Experimental Station of Pests in Xilingol Rangeland Ministry of Agriculture Xilinhot China; ^4^ Division of Plant Sciences University of Missouri Columbia MO USA

**Keywords:** courtship, mating behavior, oral sexual contact, sexual selection

## Abstract

Precopulatory courtship plays an essential role in the insemination process and influences postcopulatory behavior between males and females. Male precopulatory oral stimulation of female genitals is rare for invertebrates. Here, we describe an intriguing oral sexual courtship in a cryptic desert beetle *Platyope mongolica* Faldermann. The males repeatedly contact the female's genitals using their mouths to gain consent to mate. Furthermore, the rate at which males contact the female's genitals relates to the copulation success in a series of observations. However, interference in oral sexual contacts decreased the proportion of successful copulation. Further no‐choice tests found homosexual behavior between males with antenna removed. We report the precopulatory oral sexual behavior and its important role for copulation success in *P. mongolica* for the first time. These findings highlight the significance of oral sexual courtship in sexual selection.

## Graphical abstract

Sexual contact commonly occurs among insects (Eberhard, 1994). However, descriptions of precopulatory oral sexual contact in invertebrates remain rare and the evidence for an evolutionary function of precopulatory oral sexual contact is still lacking. We report a precopulatory oral sexual behaviour in *Platyope mongolica* Faldermann (1835), a darkling beetle typically inhabiting deserts. We provide evidence about its important role in sexual selection.

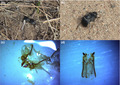

## 1 INTRODUCTION

Mating courtship is a common but important precopulatory behavior to attain female consent to copulate (Houck & Arnold, 2003; Robinson, 1982; Trail, 1985). Diverse forms of courtship exist in the animal realm such as vocalization, ritualized movements, shuttle display, and dive display (Houck & Arnold, 2003; Markow, Quaid, & Kerr, 1978). These courtships are believed to play an essential role in copulation as well as postcopulatory behavior. Although Eberhard (1994) indicated sexual contact (e.g., touching the female genital area) occurs among insects (Eberhard, 1994), oral sexual contact occurs infrequently in the animal kingdom.

A related behavior known as postcopulatory self‐sucking received more attention in the past due to its evolutionary importance in sexual selection. The literature reports this conduct in turbellaria (Schärer, Joss, & Sandner, 2004), flatworms (Janicke, Sandner, Ramm, Vizoso, & Schärer, 2016), and mammals (Bromley & Kitchen, 1974). However, this behavior is self‐oral and happens after copulation. It marks a distinct diversion from precopulatory oral sexual behavior between different sexes. Others have reported fellatio‐like activities in many mammal animals, such as bats, macaques, cheetahs, lemurs, hyenas, lions, dolphins, and bonobos (Maruthupandian & Marimuthu, 2013; Sergiel et al., 2014). Descriptions of precopulatory oral sexual contact in invertebrates remain rare. The invertebrates previously documented to exhibit oral sexual contact include Darwin's bark spider *C. darwini* (Gregorič, Šuen, Cheng, Kralj‐Fišer, & Kuntner, 2016), *Latrodectus* (Berendonck, 2003), and *Drosophila* (Spieth, 1974; Welbergen & Van Dijken, 1992; Yamamoto & Koganezawa, 2013). However, these studies only revealed a general overview of the occurrence and likely hypothetical significance of this behavior. Despite these reports, the evidence for an evolutionary function of oral sexual contact is still lacking. We know little about this precopulatory oral sexual behavior or its bearing in sexual selection.

*Platyope mongolica* Faldermann (1835) is a darkling beetle that typically inhabits deserts and serves as an indicator species for desertification (Li & Zhang, 2016). Its distribution and occurrence mirror that of the plant community, declining, or increasing in tandem with the temperate semiarid plants, such as *Artemisia ordosica* Krasch. and *Cleistogenes squarrosa* (Trinius) Keng (Li & Zhang, 2016). It is an important ecological indicator of land type succession and environmental protection. Previous studies have reported its biology (Sai‐Hong, 2001), systematics, and distribution (An, 2010; Yibin, 2012). Nevertheless, no other information documents its sexual behavior to date. We found highly frequent and conspicuous sexual behavior in *P. mongolica* during the mating season. Males followed close behind active females and fought competitors. Furthermore, *P. mongolica* engages a remarkable oral sexual courtship before copulation. However, the function and evolutionary significance of this behavior remain unknown. Whether or not this behavior is an essential stage for the success of copulation in this species is unclear. This study endeavored to uncover the understudied oral sexual behavior within this species. Findings of this study serve to make substantial contribution to sexual selection and evolution.

## 2 MATERIALS AND METHODS

### 2.1 Study site and organism

The study site is located at Scientific Observation and Experimental Station of Pests in Xilingol Rangeland, Ministry of Agriculture, Inner Mongolia, China (N43.95, E116.00) which is 120 km from Hunshandake Desert. The site represents a semiarid continental climate with a mean annual temperature of −0.1°C and annual precipitation of 350–450 mm (Qin et al., 2017). The insect species, *Platyope mongolica*, occurs from late April to middle June. It hides under grasses or detritus at night and emerges to mate during daytime. It usually shelters under plants or immersed in sand when inactive.

### 2.2 Specimens and anatomic imaging of the female's epigynum

We recorded the insect traits and mating behavior with a Canon 600D camera. We also conducted anatomic imaging of the female's epigynum using the OEM image analysis system (MD500). We chose females under two scenarios to carry out our dissection. First, female individuals without sexual contact or mating (S1), then female individuals after oral sexual contact and exposed to several unsuccessful copulation attempts (S2). We dissected and recorded the females’ genital structures under these two scenarios.

We deposited our specimens at Scientific Observation and Experimental Station of Pests in Xilingol Rangeland, Ministry of Agriculture, Inner Mongolia, China.

### 2.3 Mating duration

Our experiments started in early May. According to their natural mating procedures, we describe the mating in four steps: males pursuing females (following), oral sexual contact, mounting, and copulation. Oral sexual contact accounts for the male's first physical contact of female genitals until mounting. Mounting includes the male's attempt to climb onto the back of females until the successful insertion of its aedeagus. Copulation accounts for male's first insertion of its aedeagus into the female body until the removal. We started observing and collecting individuals during daylight at 09:00, when they emerged to mate. We collected male and female individuals that were searching for or waiting for mating partners. Females displayed their abdominal terminus upward to show interest in sexual activity. After collection, we determined whether mating occurred based on two lines of evidence: physical traits and behavioral attempts. In terms of physical evidence, we microscopically checked for male aedeagus and female genitals to see whether they remained in vaginal cavity. In terms of behavioral evidence, males showed no interest in females (e.g., not pursuant of females, and no oral sexual contact) after they successfully mated. Once females successfully mated, they continued fleeing when males pursued and females showed resistance to mating. Thus, we had confidence that our collections involved only sexually active specimens. We put one unmated male and one unmated female on sandy ground enclosed by a 1.5 m × 2 m × 0.1 m barrier under our camera surveillance. We recorded the mating processes for each pair for 6 hr (from 10:00–16:00) using the cameras. Mating usually started with the male following the female until she stopped walking and/or waited for the male. Thus, we did not count the following stage into the total mating duration. We counted the frequency (*f*) and timed the duration for each single event (*ts*) of oral sexual contact, mounting, and copulation. Accordingly, we divided the complete mating behavior into durations (*tb*) of oral sexual contact, mounting, and copulation. In total, we recorded 38 valid observations (38 pair, *N* = 38). We started recording each event from the first physical contact until the termination of final contact. We checked our video records for each mating event after witnessing all the observations in real time. We then reported frequencies (*f*) of three behaviors (oral sexual contact, mounting, and copulation), and duration of one single behavior event (*ts*), and duration of each behavior (*tb*, total events of a behavior). All of our observations were conducted on sunny days, when mating behaviors would naturally occur.

### 2.4 Mating interference and male manipulations

We hypothesized that oral sexual contact plays an important role in successful copulation. To find more evidence about this behavior in sexual selection and to make clear which parts of the male touch the female genitals, we compared the mating behaviors of *P. mongolica* among different experimental manipulations. We observed that the males used their maxillary palpi specifically to rub the female's genitals. We collected the males and females as described above. We imposed four treatments (groups) of *P. mongolica* within these mating trials. In treatment 1 (T1), we removed the males’ antennae but kept females intact. In treatment 2 (T2), we removed only the maxillary palpi of males and the females remained intact. We coated the females’ tails with petroleum jelly in treatment 3 (T3) and kept males intact. We set the normal males and females without any interference on their mating as the control group (T0). Because the mouth of this insect does not protrude, removing the lips and whole mouth would inflict serious physical damage. Therefore, we did not remove other parts of the mouth. Treatments 1 and 2 would determine which part of the males touch female genitals if there are any significant difference in mating behavior when compared to treatments 0 and 3. Treatment 3 will make clear what would happen after prevention of oral touches with the intact males and females. Therefore, treatment 3 will infer the function of oral contact when compared to treatments 1 and 2. We put one female individual and one male individual into a transparent plastic bowl (diameters: opening 20 cm; bottom 10 cm), each filled with 2 cm of sand at the bottom. Individuals reluctant to mate for more than an hour after another individual had touched it were replaced. Each treatment had 5 replicates (10 individuals, 5 males, and 5 females). In total, we made 20 valid pair‐mating trials. We checked the number of individuals that successfully copulated after they finished mating. We dissected the bodies of all mated individuals after finishing the experiments. Males usually left the spermatophore in female's body if the copulation completed successfully.

We also observed male–male touches during field observations. To ensure the mating attempts were not by accident, we also performed the same treatments as the previous four treatments but using only males. We recorded all the processes of mating pairs during the investigation. We recorded the frequency and duration (*ts*, *tb*) of oral sexual contact, mounting duration, and copulation duration following the same procedure as described above.

### 2.5 Statistical analysis

The frequency and duration of oral sexual contacts, mounting, and copulation were compared using *t* test. The difference between copulation duration under different treatments was analyzed using ANOVA. Regressions on the relationship between the frequency/duration of oral sexual contacts and mounting/copulation were conducted in R using a linear model.

## 3 RESULTS

### 3.1 Characteristic of mating behavior in *P. mongolica*

*Platyope mongolica* possess chewing mouthparts, and they feed on plant leaves. Their mouthparts project down, lack mandibles, and galeae, but contain short feeler‐like palps attached to the maxillae. Its maxillary and labial palpi function both in feeding behavior by bringing food into the mouth and in sexual behavior by stimulating the female's genitals.

**FIGURE 1:**
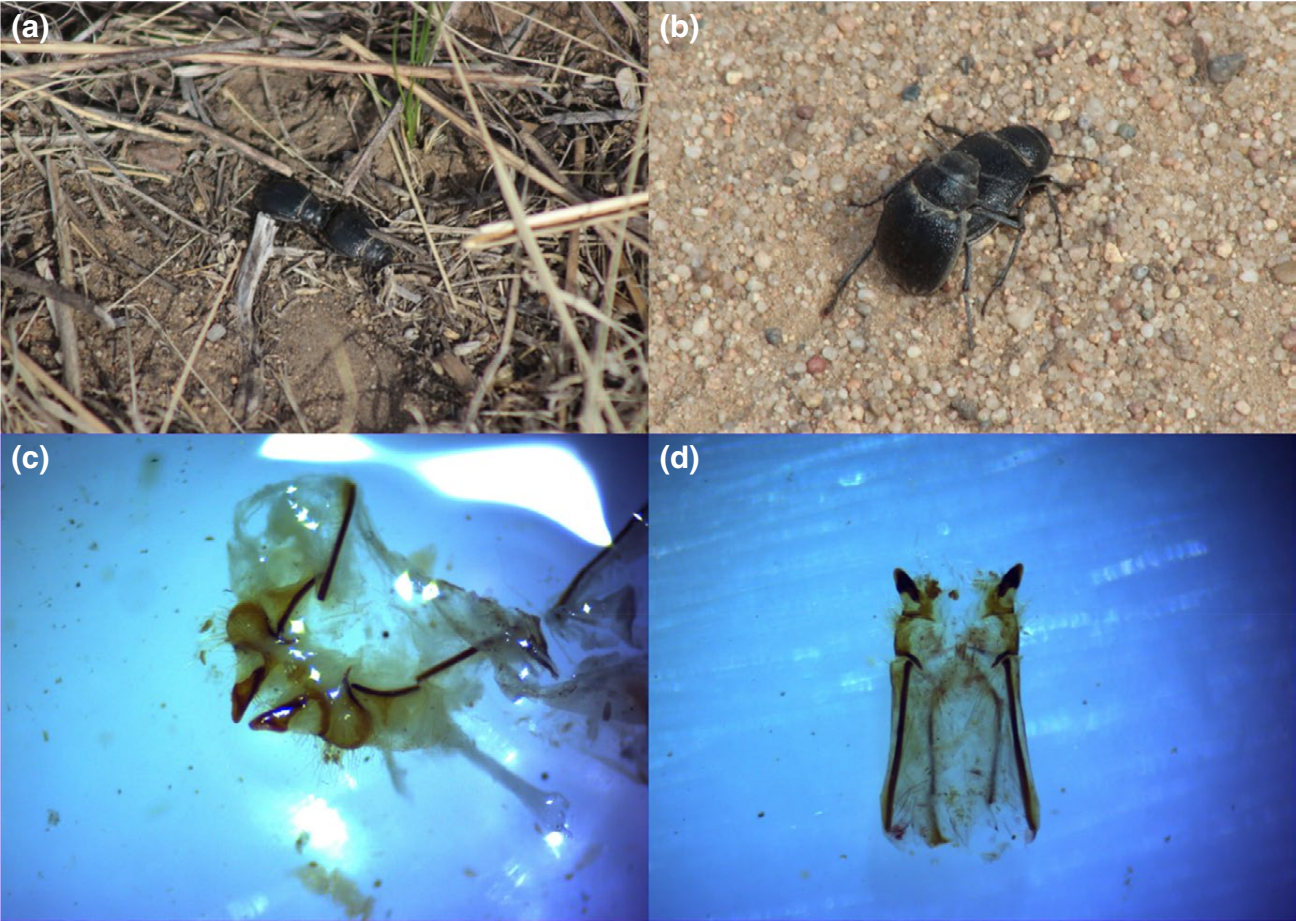
*Platyope mongolica* mating and anatomic imaging of female's epigynum. (a) Oral sexual contacts between male and female. (b) Male–female copulation. (c) Epigynum from individuals without any oral sexual touches, in posterior view under anatomic imaging. (d) The epigynum from individuals after oral sexual contacts (dorsal view)

Sexual encounters start with the females waiting for pursuant males. Females usually stop moving and prostrate their heads as they extend their abdominal terminus higher to submit to mating. Males search and pursue females until the females stop moving. The remarkable courtship before copulation is that males repeatedly rub their maxillary palpi on the female's genitals before an attempt to copulate (Figure 1a). However, if the female is not satisfied with the male's performance, the female usually runs away. In that case, the male continues pursuit and genital rubbing until another attempt to copulate. This procedure repeats until the male successfully inserts his aedeagus or the female rejects the copulation (Figure 1b).

Anatomic imaging shows the female's short pair of genital plates with eye­like anterior margins. Notably, the vulva is narrow and anterior copulatory duct is long (Figure 1c, d). The epigynums are inward‐turned, and the vulva is closed before copulation without any oral sexual contacts (Figure 1c). However, the epigynums are outward‐turned and the vulva opens after the oral sexual contact (Figure 1d). The normal status of sharp genital plates with narrow vulva likely results in low possibility of unexpected insertion. In contrast, the opening epigynums likely make the insertion easier.

### 3.2 Mating duration

**FIGURE 2:**
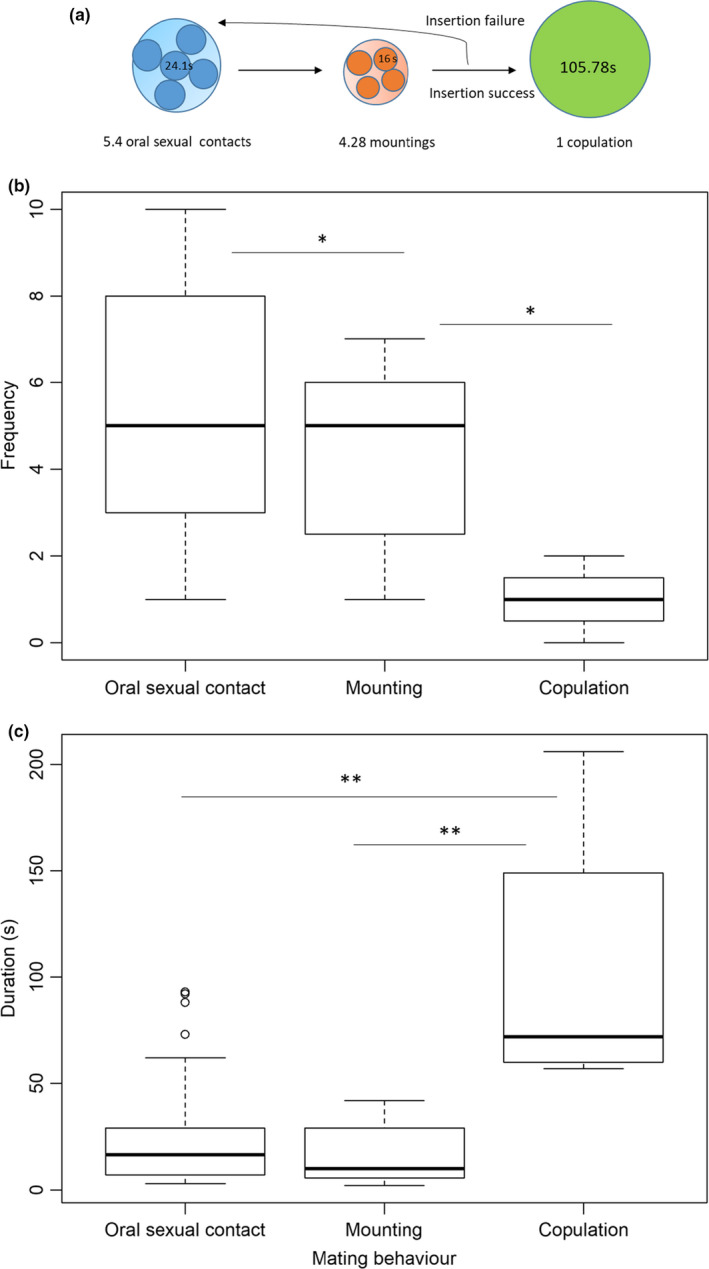
The frequency and duration of each mating process from observations under natural conditions (without manipulation). (a) The mating procedure and behavioral transitions to complete copulation. The arrow indicates the behavioral transition. The size of the circle indicates the average time spent on that behavior until a successful copulation event happened. The number of small circles within the big circle indicates the number of single events (frequency), and the values indicate the duration of a single event. (b) The frequency of different mating behaviors. (c) Average time spent on a single behavior event (*ts*). Error bars in B and C represent the standard deviations. The lines with asterisks in B and C indicate the comparison between two behaviors. *P* value adjustment method: Holm. * indicates the significance at *p* < .05, and ** indicates the significance at *p* < .001 tested using *t* tests

Among all the mating processes (oral sexual contact, mounting, and copulation), the frequency (*f*) of oral sexual contact affects the variance in successful copulation the most (*F* = 250, *p* = 2.56e−07). Generally, the frequency of mounting reflects the number of unsuccessful attempts to copulate (insertion failure). Mating pairs underwent 5.4 oral sexual contacts lasting about 24 s each time. Mating pairs then exhibited 4.28 mountings of about 16 s per mount to achieve one copulation (Figure 2a). The frequency of oral sexual contact is significantly higher than the frequency of mounting and copulation (mounting versus copulation *p* = .049, *N* = 38; oral sexual contact versus copulation, *p* = .012, *N* = 38; Figure 2b). However, duration of a single copulation event lasted longer than the duration of a single oral sexual contact event (oral sexual contact versus copulation, *p* = 3.4e‐07, *N* = 38; mounting versus copulation *p* = 3.9e−08, *N* = 38; Figure 2c).

### 3.3 Mating interference

**FIGURE 3:**
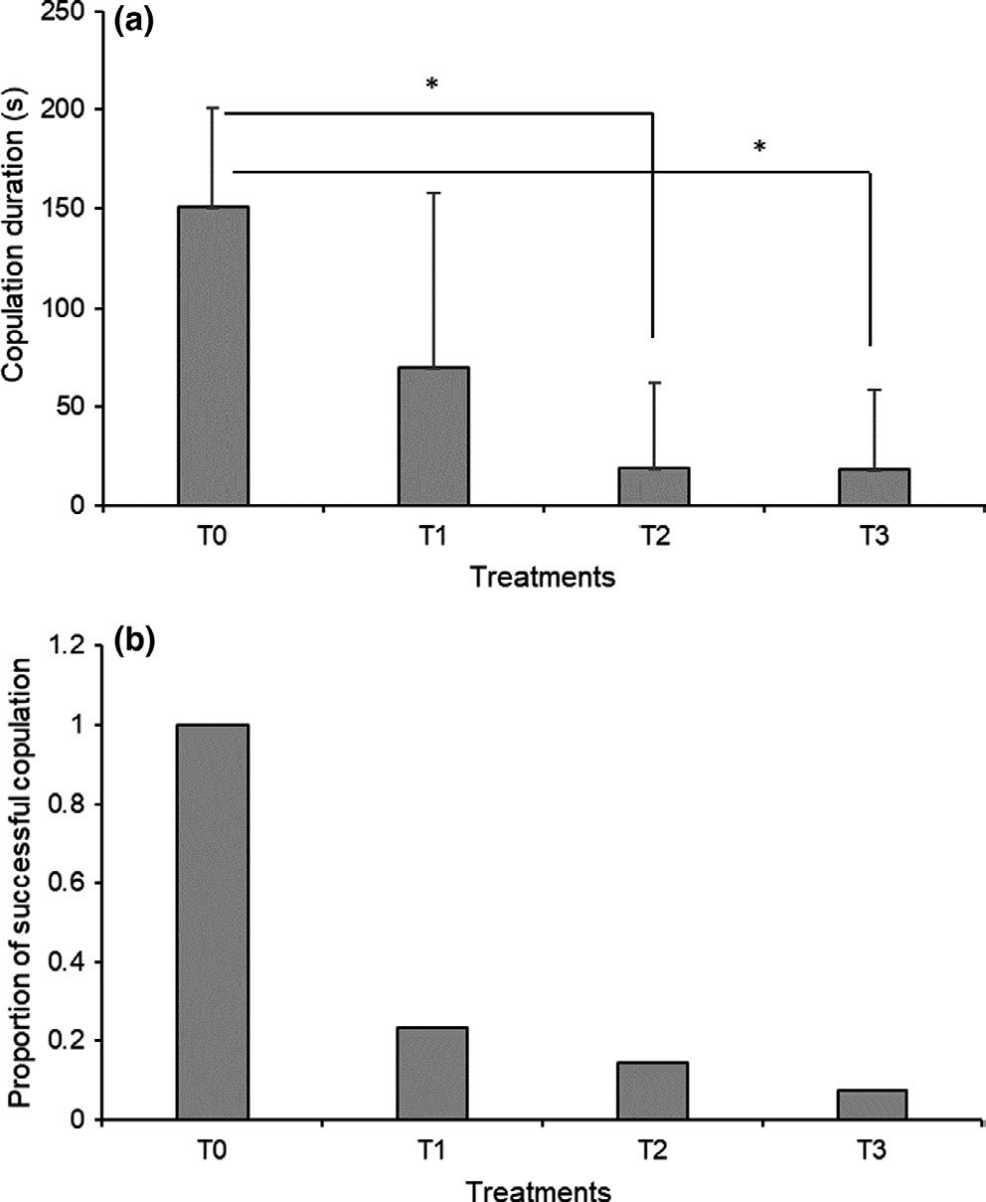
(a) Copulation duration (*tb*) under different treatments. Error bars indicate the standard deviations. (b) Proportion of successful copulations happening under different treatments. T0 indicates treatment with intact males and females, T1 indicates treatment with normal females mixed with males lacking antennae, T2 indicates treatment with normal females mixed with males lacking maxillary palpi, and T3 indicates treatment with normal males mixed with females whose tails were coated with petroleum jelly. * indicates the significance at *p* < .05 tested using *t* tests

To make clear which parts of the male touch the female genitals, we compared the mating behaviors of *P. mongolica* among different experimental manipulations [with the antennae of males removed (T1), the maxillary palpi of males removed (T2), the females’ tails coated with petroleum jelly (T3), and intact males and females (T0)]. Results showed that copulation duration significantly decreased after removal of males’ maxillary palpi (*p* = .03, *N* = 27) and coating females’ tails with petroleum jelly (*p* = .03, *N* = 27; Figure 3a). Removal of males’ antennae did not decrease copulation duration with females (*p* = .33, *N* = 27; Figure 3a). However, all the interference treatments (T1‐T3) have a significant decrease in the rate of successful copulation (Figure 3b). Interestingly, the attempted same‐sex behavior occurred among males missing antennae in 80% of the trails (under the same condition with T1 but using only males), indicating their role in identifying partners. These results support our observation that the male rubs the female's genitals using their mouthparts, mainly maxillary palpi, as there are no protrusive lips or other galeae in this species. Meanwhile, differences in copulation duration between T0, T1, T2, and T3 indicates that prevention of oral sexual contact leads to significant interference in copulation, which could potentially lead to an increase in the rate of copulation failure (Figure 3b). This outcome suggests the essentiality of this trait in mating evolution.

### 3.4 Relationship between oral sexual contact and copulatory success

**FIGURE 4:**
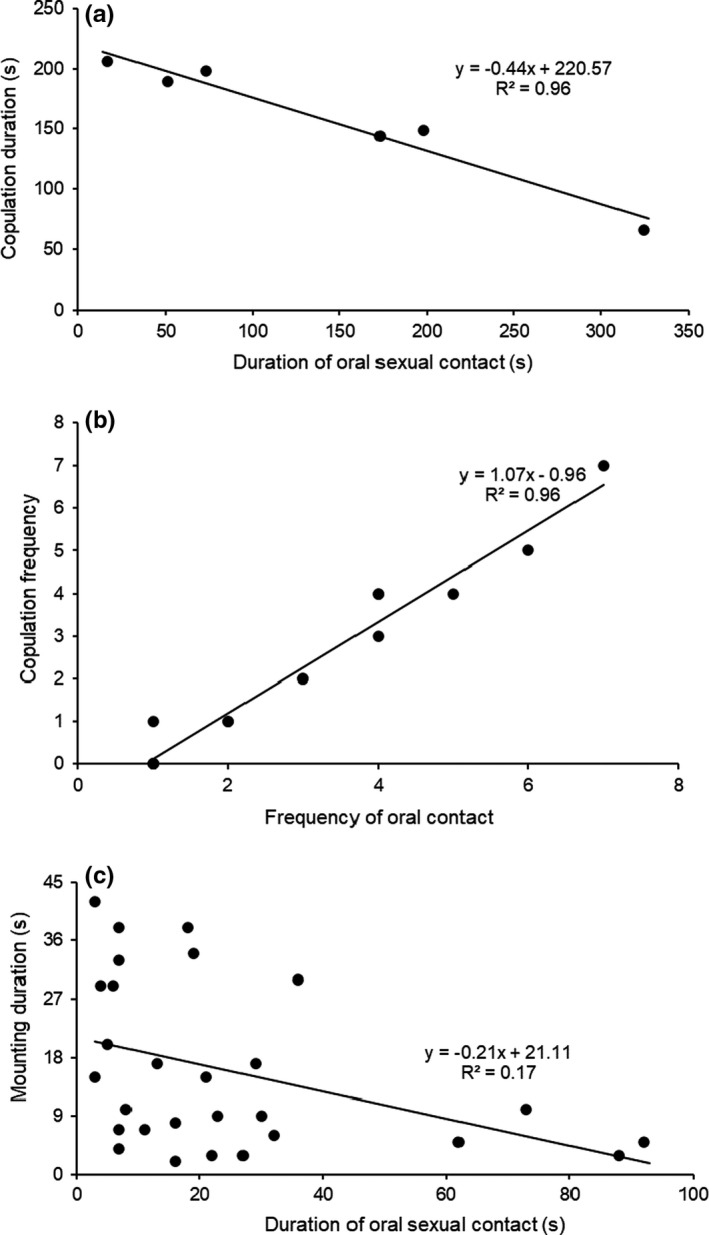
(a) The relationship between copulation duration (*tb*) and duration of oral sexual contact. (b) The relationship between copulation frequency and frequency of oral sexual contact. (c) The relationship between mounting duration and duration of oral sexual contact

Further analysis showed the relationship between oral sexual contact and copulatory success. Investment in duration of oral sexual contact shortened copulation duration (*R* ^2^ = 0.96, *F* = 86, *p* < .001, *N* = 6, 6 successful copulations in field observations; Figure 4a). We also found that copulation frequency correlated positively with the frequency of oral sexual contact from manipulation experiments (Figure 4b; *R^2^* = 0.96, *p* = 1.99e‐10, *N* = 15). Consistently, mounting duration also decreased linearly with the increase in duration of oral sexual contact (Figure 4c; *R* ^2^ = 0.17, *F* = 5.17, *p* = .032, *N* = 28). It is worth noting that increasing oral sexual contact is correlated with increased copulation attempts, but also a decrease in copulation duration. The shorter a male spends on oral sexual contact, the longer a male spends on copulation attempts, and the more likely it is to be unsuccessful (Figure 4).

## 4 DISCUSSION

Previous studies reported interesting postcopulatory self‐oral sexual contacts in the marine turbellarian *Macrostomum lignano* (Schärer et al., 2004). The worm places its pharynx over its own female genital opening and appears to apply suction (Schärer et al., 2004; Schärer, Littlewood, Waeschenbach, Yoshida, & Vizoso, 2011). Although it was proposed that this postcopulatory behavior may be an adaptation to manipulate sperm or secretions received during copulation (Schärer et al., 2004), whether precopulatory oral sexual contacts between different sexes can contribute to copulatory success remains unclear and rarely studied. Therefore, it is crucial to investigate the evolution of this trait. In this study, we reported an oral sexual behavior correlated with increased copulatory success in a cryptic desert beetle *P. mongolica*. We provide further evidence that the oral sexual behavior promotes copulation success, indicating this behavior may be sexually selected.

Courtship consists of a series of steps, in general, including pursuit, oral sexual contact, mounting and copulation, sperm transfer, and postcopulation behaviors. Each step is important, and often, the individuals cannot move to the next step until the previous step has been correctly performed. If a male does not have a good performance in a certain step and moves too quickly to the next step, it most likely will need to return to a previous step again. Therefore, each step is crucial, and each must be accomplished to successfully reach the end (sperm transfer). We observed that copulation accounted for the longest average time (*ts*) during mating (Figure 2a–c). However, achieving copulation is not easy in this species. On average, one copulation (successful insertion) usually required at least as much time or more than that invested in oral sexual contacts. The data showed 5 experiences in oral sexual contact after 4 unsuccessful copulation attempts before the successful insertion (Figure 2). Our evidence showed that sharp genital plates and narrow vulva in this species prevent the probability of smooth insertion absent of proper oral sexual stimulation (Figure 1c). Investment in oral sexual contact promotes the successful insertion by stimulating the opening of the vulva (Figure 1d). Oral sexual contact not only promotes mating success but also reduces the unsuccessful insertion cost and copulation duration (Figures 3 and 4).

A study reported a similar behavior in *Μantichorula semenowi* Reitter, which is in the same family as *P. mongolica*. However, this behavior was described as “the male rubs the female's tail using antenna and head” (Zhang, Yu, & Ren, 2003), quite different from our observations. To clarify which male parts touch the female genitals, we compared the mating behaviors of *P. mongolica* among different experimental manipulations. Our results showed that the male rubs the female's genitals using their maxillary palpi. This is an interesting finding and clarification of oral sexual behavior in *P. mongolica*. Nerveless, our study also showed that *P. mongolica's* antennae play an important role in identifying mating partners. The antennae contain large numbers of chemical receptors that enable insects to detect mating partners via pheromones, look for food, and find oviposition habitats through volatile compounds (Carraher et al., 2015). Even though removing the males’ antennae did not decrease copulation duration between different sexes, males attempted the same‐sex behavior when they were put together. Previous studies have found that the sex pheromone receptor in the antennae of a male is the minimally sufficient determination factor that triggers initiation of orientation behavior toward a potential mate (Sakurai et al., 2011). In agreement with these reports, our study suggests that the antennae fulfill a common fundamental role in enabling insects to identify a mating partner of the opposite sex in sexual behavior.

Oral sexual behaviors, in particular cunnilingus‐like behaviors, are still rarely studied. A most recent case about oral sexual contact/encounter is Darwin's bark spider *C. darwini*, whose copulation involves a series of extreme sexual behaviors, such as sexual cannibalism, genital mutilation, male preference for teneral females and emasculation, and remarkably, the oral sexual encounters (Gregorič et al., 2016). However, testing the function of oral sexual contact in Darwin's bark spider seems to be impossible because this behavior not only happens before copulation but also in between bouts and after copulation (Gregorič et al., 2016). Another case of this similar behavior was documented in the fruit fly (Spieth, 1974), whose mating includes males orally stimulating female genitals before copulation (Hall, 1979; Yamamoto & Koganezawa, 2013). However, it was reported that this licking behavior only induced changes in male mating speed without any effects on female’ s sexual behavior (Welbergen & Van Dijken, 1992). Evidence about the evolution of precopulatory oral sexual behavior in insects is still extremely lacking.

Previous studies have indicated that precopulatory mating behavior can directly increase mating success by influencing female's mate choice and mating continuity through intersexual interactions (Eberhard, 1994; Elias, Sivalinghem, Mason, Andrade, & Kasumovic, 2014; Neff & Svensson, 2013; Shuker et al.., 2002). We show that precopulatory oral sexual contact is an important courtship during mating in this species and could lead to sexual selection: It influences the success of copulation. We provided important evidence to demonstrate the role of oral sexual contacts in successful copulation. First, in the manipulation experiments, interference of oral sexual contact led to significant decrease in copulation duration (*tb*) and success rate of copulation (Figure 3a, b). This means selection directly acts on oral sexual contact. Second, increase in duration of oral sexual contact shortens copulation duration (Figure 4a). A study showed that fellatio by females (females licked the male's penis) could prolong the copulation time in fruit bats (Tan et al., 2009). Lots of studies indicated that prolonged copulation duration in insects reflected the higher mating quality and the higher likelihood of fertilization success (Andrés & Rivera, 2000; Nuyts & Michiels, 1993; Wang, Yang, & Hedderley, 2008). However, longer copulation does not always represent more benefits to a species inhabiting in dry desert and mating upon the bare sandy ground. Rather, the longer a male spends on copulation attempts, the more likely it is to be unsuccessful. Therefore, investment in oral sexual contact decreases the copulatory cost, particularly for species undergoing heat stress and prey risk during mating. Third, mounting duration also decreases with the increase in duration of oral sexual contacts (Figure 4c). This indicates there is a tradeoff between oral sexual contact and insertion failure/success. Investment in oral sexual contact promotes the insertion success, thus increasing the copulation efficiency throughout the mating process.

This evidence shows that an increase in the frequency of oral sexual contact increases the successful rate of copulation. In addition, increased duration of oral sexual contact reduces the insertion cost (e.g., decrease in mounting duration) as well as total copulation duration. From an evolutionary perspective, this is very important for beetles living in harsh environments, especially in semiarid desert. As recorded in Zhang et al. (2003), the morphology of *P. mongolica* makes it difficult to copulate: Male and female bodies present perpendicularly to each other during copulation (Zhang et al., 2003). In *P. mongolica*, males could benefit from spending a significant amount of time on oral sexual contact if this restricts the female's access to alternative mates. Thus, it increases the likelihood of the current male mating success. Our study also reveals that a large investment in precopulatory oral sexual contact is the cost for successful copulation in this species.

Meanwhile, the results from interference experiments seem to contradict the conclusion that investment in oral stimulation shortened copulation. It is worth noting that we reported the duration of a behavior (*tb*) in experimental manipulations. There were a low number of successful copulations, and most of them failed to copulate in males with damaged palpi (T2) and females covered with vaseline (T3). All the interference treatments (T1‐T3) have a significant decrease in the frequency of copulation and thus a significant decrease in the rate of successful copulation (Figure 3b). This is why the duration of copulation is shorter in groups with manipulations. To enhance the ability of successful reproduction, males usually balance their investment in traits that can increase their successful access to mates (Barrett, Evans, & Gasparini, 2014), and any traits that can increase the likelihood of copulation and insemination will benefit the reproduction of this species. Consequently, precopulatory oral sexual contacts could facilitate and promote process for copulation. The specialized phenotypes, including oral sexual contact and the special genital structure, indicate evolution through sexual selection in this species.

This is the first time that precopulatory oral sexual behavior in the desert darkling beetle, *P. mongolica,* is reported. We investigated the role of this oral sexual behavior in copulation. The results suggest that precopulatory oral sexual contact, an important phase of mating latency in *P. mongolica*, plays an important role in copulation. We confirm that precopulatory oral sexual contact is a form of investment for successful copulation. Our study adds to a new understanding of the evolutionary significance of precopulatory oral sexual behavior in insects, making substantial contributions to sexual selection and evolution.

## CONFLICT OF INTEREST

The authors state that there is no conflict of interest.

## AUTHOR CONTRIBUTION

**Xinghu Qin:** Conceptualization (lead); Data curation (lead); Formal analysis (lead); Investigation (lead); Methodology (lead); Resources (lead); Software (lead); Supervision (lead); Validation (lead); Visualization (lead); Writing‐original draft (lead); Writing‐review & editing (lead). **Jinshu Yang:** Conceptualization (equal); Data curation (equal); Investigation (equal); Methodology (equal); Resources (equal); Validation (equal). **Jingchuan Ma:** Data curation (equal); Formal analysis (equal); Investigation (supporting); Methodology (supporting); Resources (supporting); Software (supporting). **T. Ryan Lock:** Data curation (supporting); Formal analysis (equal); Resources (supporting); Validation (supporting); Writing‐review & editing (equal). **Guangjun Wang:** Conceptualization (equal); Data curation (equal); Funding acquisition (equal); Investigation (equal); Project administration (equal); Resources (equal); Supervision (equal); Writing‐original draft (equal). **Zehua Zhang:** Data curation (equal); Formal analysis (equal); Funding acquisition (equal); Methodology (equal); Project administration (equal); Resources (equal); Supervision (equal); Writing‐review & editing (equal).

## Data Availability

The data used in our study now are stored at Open Science Framework Archive (https://osf.io/mtqah/?view_only=c6c5a40574b144a1879434f13efc4c99).
